# Prediction of Performance in a Short Trail Running Race: The Role of Body Composition

**DOI:** 10.3389/fphys.2019.01306

**Published:** 2019-10-16

**Authors:** José Ramón Alvero-Cruz, Verónica Parent Mathias, Jerónimo Garcia Romero, Margarita Carrillo de Albornoz-Gil, Javier Benítez-Porres, Francisco Javier Ordoñez, Thomas Rosemann, Pantelis T. Nikolaidis, Beat Knechtle

**Affiliations:** ^1^Faculty of Medicine, Andalucía Tech, University of Málaga, Málaga, Spain; ^2^Faculty of Medicine, University of Cádiz, Cádiz, Spain; ^3^Institute of Primary Care, University of Zurich, Zurich, Switzerland; ^4^Exercise Physiology Laboratory, Nikaia, Greece; ^5^Medbase St. Gallen Am Vadianplatz, St. Gallen, Switzerland

**Keywords:** maximal oxygen uptake, fat mass, skeletal muscle mass, short trail running, performance prediction

## Abstract

The aim of the present study was to examine the role of the classical physiological model of endurance running performance – maximal oxygen uptake (VO_2_max), %VO_2_max at ventilatory thresholds (VT), work economy, lactate levels, and body composition on the prediction of short trail running performance. Eleven male trail runners (age 36.1 ± 6.5 years, sport experience 6.6 ± 3.8 years, and mean ± standard deviation) were examined for fat mass and skeletal muscle mass, and performed a graded exercise test to measure VO_2_max, vVO_2_max, and VT. Also, they participated in a short 27 km trail run with a positive elevation of +1750 m. Age, years of training and skeletal muscle mass did not correlate with race time (*P* > 0.05), and fat mass and body mass index (BMI) showed significant correlations with race time (*P* < 0.05). Heart rate, velocity and VT1 and VT2 were not associated with race time (*P* > 0.05). Only vVO_2_max (*P* = 0.005) and VO_2_max (*P* = 0.007) is correlated to race time. Multiple regression models for VO_2_max accounted for 57% of the total variance. The vVO_2_max model variable accounted for 60% and the fat mass model for 59.5%. Finally, the combined VO_2_max and fat mass model explained 83.9% of the total variance (*P* < 0.05 in all models). The equation for this model is “race time (min) = 203.9956−1.9001 × VO_2_max + 10.2816 × Fat mass%” (*R*^2^ = 0.839, SEE = 11.1 min, and *P* = 0.0007). The classical variable VO_2_max together with fat mass percent are two strong predictors for short trail running performance.

## Introduction

Trail races typically involve running over short to long or extreme distances on irregular terrain with large positive and negative elevation changes (ITRA, 2019).

Long-distance running performance is usually predicted by VO_2_max, with its fractional component (%VO_2_max), ventilatory and lactate thresholds, and running economy considering this a “classical model” of assessment (Sjodin and Jacobs, 1981; Morgan et al., 1989; Midgley et al., 2007; McLaughlin et al., 2010; Barnes and Kilding, 2015; Vernillo et al., 2017b; Ehrström et al., 2018).

Due to the characteristics of mountain race routes, different physiological, muscular and biomechanical demands are involved, determined by the constant uphill and downhill sections of terrain, resulting in different fatigue patterns, and providing the opportunity to evaluate other factors related to trail running (Giandolini et al., 2016; Balducci et al., 2017; Vernillo et al., 2017a). For this same reason some authors have looked for other methods to improve performance prediction based on the application of different graded exercise test (step, ramp, and trail) to observe what type of protocol improve the prediction (Scheer et al., 2018b).

There are other prediction models for trail running as markers of muscle function and fatigue, such as maximum voluntary isometric contraction, countermovement jumping, muscle stiffness, muscle pain, and the energy cost of running (Balducci et al., 2017; Ehrström et al., 2018) and finally improve prediction of trail running performance including not only the classical model (VO_2_max and running economy) but also variables such as vertical velocity, kinetic data using pressure templates, space-time data combining kinematic, and force data (Björklund et al., 2019).

Other factors as body composition (adequate fat mass percent and lean body mass) are related to performance in trail runners (Hoffman, 2008). Other studies carried out in ultramarathon mountain runners include prediction models based on body fat and BMI, the maximal power values from exercise testing and VO_2_max, both at level the aerobic and anaerobic thresholds (Fornasiero et al., 2018).

The relationship between performance and body composition in endurance athletes resides in low levels of adiposity as more muscle effort is required to accelerate the legs, and consequently energy expenditure at the same speed would be higher. Similarly, adequate muscle masses are required that do not increase body weight (Mooses and Hackney, 2017). The study of these factors is of great interest to athletes, coaches and physiology researchers to find, through specific physiological evaluations both in the laboratory and in the field, different variables to predict performance and thus improve training plans and competition results.

The first objective of this study was therefore to identify the predictive power of the classical variables for determining in a short-duration trail running performance. These factors together with the inclusion of body composition variables such as fat mass and muscle mass, maintaining the hypothesis that these would improve predictive ability. The second objective was to determine whether the classical variables are adequate to explain performance in trail races and to provide a useful tool for athletes and coaches to monitor training and improve performance.

## Materials and Methods

### Ethical Approval

All the participants were informed of the procedures and signed an informed consent prior to the start of the experimental protocol. The protocol used in this study was approved by the Ethics Committee of the University of Málaga (2013-EMEFYDE-006) and was in accordance with the Declaration of Helsinki (JAMA, 2013).

### Participants

The laboratory study involved eleven trained male trail runners. All participants were informed of the procedures and signed an informed consent prior to the start of the experimental protocol.

### Experimental Design

A retrospective study was performed based on physiological laboratory assessments in February 2013, and the trail race was held at the beginning of March of the same year. For collection of the independent variables associated with the different assessments, all the participants underwent a body composition assessment using anthropometry and an incremental graded exercise test with analysis of expired air, as metabolic equations is the difference in fractions of inspired and expired O_2_ and CO_2_ 2 weeks prior to the race. Maximum heart rate was used for quality control of maximal effort exerted during the test.

### Procedures

#### Anthropometric Assessment

All measurements were conducted after a 12 h fast. Weight was measured on a SECA 813 electronic scale (Hamburg, Germany) with an accuracy of 0.1 kg, and height was measured using a wall-mounted SECA 216 stadiometer (Hamburg, Germany) with an accuracy 0.1 cm. Skinfolds were measured in triplicate at the following sites: triceps, subscapular, biceps and iliac crest, with a Holtain skinfold caliper (Holtain, Crymych, United Kingdom) computing the means for subsequent calculations. Percentage of body fat was estimated with the Faulkner equation (Faulkner, 1968) as follows:

Percentfatmass=0.153×Σ(triceps,subscapular,biceps,   andiliaccrestskinfolds)+5.786.

Skeletal muscle mass with Lee’s equation (Lee et al., 2000) as follows:

SM = Ht × (0.00744 × CAG^2^ + 0.00088 × CTG^2^ + 0.00441 × CCG^2^) + 2.4 × sex −0.048 × age + 7.8.

Using corrected arm, thigh, and calf girth measurements taken with a Holtain anthropometric tape (Holtain, Crymych, United Kingdom). All measurements were collected following the standardized procedures of the International Society for Advancement in Kinanthropometry (Marfell-Jones and Olds, 2006). The technical error of measurement (TEM) of the technician level 3 anthropometrist was less than 3% for skinfolds and 1% for the rest of the anthropometric measurements. TEM was calculated as follows:

Absolute TEM = √Σ di^2^/2n, where: Σd2 = sum of deviations raised to the second power, n = number of volunteers measured and i = the number of deviations.

#### Laboratory Test

All participants underwent a maximal incremental exercise test to determine VO_2_max, as well as respiratory exchange variables such as carbon dioxide output (VCO_2_), end-tidal oxygen tension (PetO_2_), end-tidal carbon dioxide tension (PetCO_2_), ventilation, and respiratory exchange ratio (RER). The expired gases were measured breath by breath and recorded in an Ultima CPX metabolic measurement cart (MedGraphics, Saint Paul, MN, United States). The system was automatically calibrated before each test, with a calibrated gas mixture (O_2_: 15% and CO_2_: 5%) and volume analyzer using a precision 3L syringe (MedGraphics, Saint Paul, MN, United States). according to the manufacturer’s instructions. Heart rate was recorded using a telemetric electrocardiography device (X-Scribe, Mortara, Milwaukee, WI, United States) connected to the system. Aerobic (VT1) and anaerobic (VT2) thresholds were determined using Skinner and McLellan (1980) guidelines.

**FIGURE 1:**
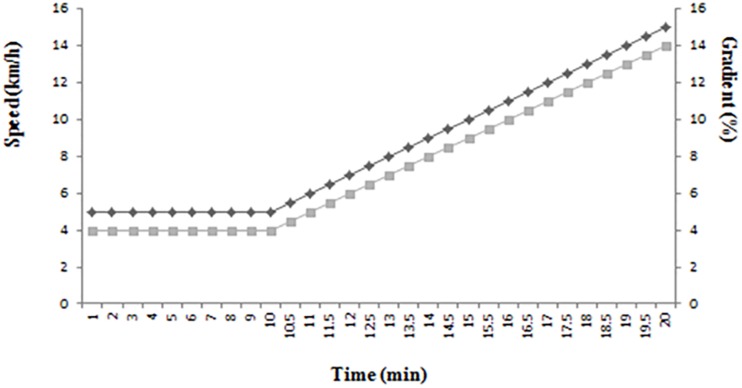
Exercise test protocol.

The participants ran on a motorized treadmill (Ms Medisoft 870, Medisoft, Italy) according to the following protocol: After a 10 min warm up at 5 km/h with a constant gradient of 4%, the test began at 5.5 km/h with speed increments of 0.5 km/h/0.5% gradient/min to volitional exhaustion (Figure 1). The test was considered maximal when: RER > 1.1 or there was an increase of less than 2.1 ml/kg/min in VO_2_ between the two stages, or when a range ±10 beats/min of the maximum predicted heart rate was reached, without these being excluding requirements, according to ACSM Guidelines for exercise testing and prescription (American College of Sports Medicine, 2014). The velocity corresponding to VO_2_max (vVO_2_max), was defined as the minimum velocity at which VO_2_max is reached (Billat et al., 1996). All the participants received verbal encouragement from the investigators to give their maximum possible effort. The percentage with respect to the theoretical heart rate (220-age) was calculated from the heart rate values.

#### Lactate

At the end of the race within the first minute, a 0.5 μL blood sample taken from the earlobe was obtained for the measurement of blood lactate concentration, using an electrochemical device (Lactate Pro LT-1710, Arkray, Japan). The coefficient of variation of the analyzer used is 3%. The blood lactate analyzer was checked before using a manufacturer calibration strip.

#### Trail Race

All participants performed the “Los Guájares Trail” which took place in Granada (Spain) in March of 2013. The course was 27 km long with a total positive elevation of +1750 m (ITRA Category XS).

### Statistical Analyses

The data are presented as means and standard deviations. Normality was analyzed using the Shapiro-Wilk test. Since all the variables were normally distributed, an association analysis between variables was performed using Pearson product-moment correlation coefficients. The following criteria were adopted to interpret the magnitude of the correlations: *r* ≤ 0.1, trivial; 0.1 < *r* ≤ 0.3, small; 0.3 < *r* ≤ 0.5, moderate; 0.5 < *r* ≤ 0.7, large; 0.7 < *r* ≤ 0.9, very large; and *r* > 0.9, almost perfect (Hinkle et al., 2003). Variables significantly associated with race time in the trail race were included in a stepwise multiple regression analysis to estimate the predictors of race time (dependent variable) from two blocks of independent variables (from the laboratory and the anthropometric assessment). Partial correlation (covariates, fat mass percent, BMI, and weight) procedures were used to evaluate the relationships between race time and VO_2_max.

The level of significance in all cases was set at P < 0.05. The statistical analysis was performed on MedCalc Statistical Software version 19.0.3 (MedCalc Software bvba, Ostend, Belgium^1^).

## Results

The descriptive values of the runners relative to the variables of age, years of training, and body composition are shown in Table 1.

**TABLE 1:** Descriptive data for runners and body composition variables.

**Variable**	**Mean**	**SD**	**Min**	**Max**
Age (years)	36	6.5	23.00	54.00
Training (years)	6.6	3.8	1.00	15.00
Running race time (min)	186	24.75	145	215
Lactate (mmol/L)	6.32	2.77	2.9	11.7
Weight (kg)	68.09	6.35	58.20	76.00
Height (cm)	173.21	7.61	163.00	186.00
BMI (Kg/m^2^)	22.67	1.62	20.45	25.90
Fat mass (%)	9.96	1.35	8.41	12.79
Fat mass (kg)	6.82	1.34	5.10	9.34
Skeletal muscle mass (%)	46.37	2.66	40.10	50.22
Skeletal muscle mass (kg)	31.48	2.36	27.77	35.87

*BMI, body mass index.*

Table 2 shows the variables obtained in the laboratory including heart rate, velocity and oxygen consumption at the aerobic (VT1), anaerobic (VT2), and maximum thresholds.

**TABLE 2:** Variables measured in laboratory test.

**Variable**		**Mean**	**SD**	**Min**	**Max**
HRVT1	bpm	147	8	137.00	160.00
HRVT2	bpm	165	7	155.00	176.00
HRmax	bpm	181	8	168.00	195.00
vVT1	km/h	8.07	0.47	7.00	9.00
vVT2	km/h	9.75	0.55	8.50	10.50
vVO_2_max	km/h	11.39	0.63	10.50	12.50
VO_2_VT1	ml/kg/min	43	6	35.00	53.80
VO_2_VT2	ml/kg/min	58	5	51.90	68.50
VO_2_max	ml/kg/min	67	7	55.10	80.20

*HR, heart rate; VO_2_, oxygen consumption; VT1, ventilatory threshold 1; VT2, ventilatory threshold 2.*

Correlations between body composition variables and race time are shown in Table 3. There was no significant correlation for training years, age and skeletal muscle mass (*P* > 0.05). The remaining variables (fat mass and BMI) showed a significant correlation with race time (*P* < 0.05).

**TABLE 3:** Pearson product-moment correlation coefficients between body composition variables and race time.

		**Training years**	**Age**	**Fat mass %**	**Fat mass kg**	**BMI**	**SMM kg**
Race time (min)	*r*	0.349	0.36	0.772	0.711	0.6	0.34
	*P*	0.293	0.28	0.0054	0.0142	0.05	0.3067

*BMI, body mass index; SMM, skeletal muscle mass.*

**FIGURE 2:**
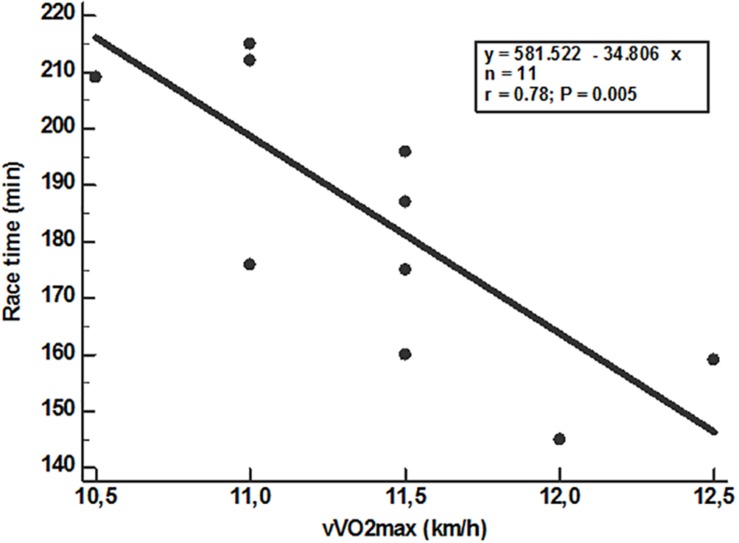
Correlation between race time and vVO_2_max.

**FIGURE 3:**
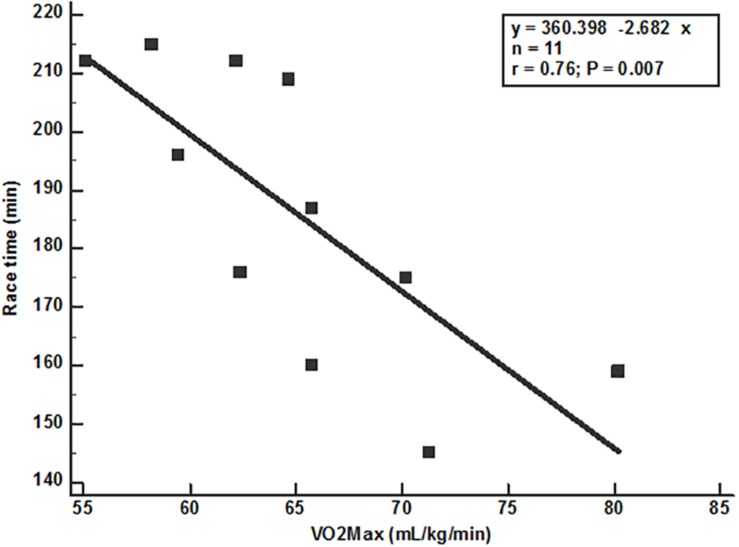
Correlation between race time and VO_2_max.

Table 4 shows the correlations between race time and the laboratory variables at VT1, VT2 and the maximum, velocities, and oxygen consumption at VT1 and VT2 were not significant (*P* > 0.05). Only vVO_2_max (*P* = 0.005) and VO_2_max (*P* = 0.007) showed significant correlations with time race (Figures 2, 3). VO_2_max and fat mass percent (*r* = −0.39 and *P* = 0.23) do not correlate significantly. However, race time show significant inverse correlation with VO_2_max (*r* = −0.78 and *P* = 0.02) but is not influenced by covariates as fat mass percent, BMI, or weight.

**TABLE 4:** Pearson product-moment correlation coefficients between laboratory variables and race time.

		**HRVT1**	**HRVT2**	**HRmax**	**vVT1**	**vVT2**	**vVO_2_max**	**VO_2_VT1**	**VO_2_VT2**	**VO_2_max**
Race time (min)	*r*	0.471	0.262	0.092	−0.329	−0.505	−0.776	−0.272	−0.428	−0.757
	*P*	0.1438	0.4369	0.787	0.3224	0.1133	0.005	0.4184	0.1889	0.007

*HR, heart rate; v, velocity; VO_2_, oxygen consumption; VT1, ventilatory threshold 1; VT2, ventilatory threshold 2.*

Lactate values at the end of the competition were not associated with performance (*P* > 0.05).

The results of the multiple regression models analyzed are presented in Table 5. The model for VO_2_max explained 57% of the total variance. The model for the vVO_2_max variable explained 60%. The fat mass percent model explained 59.5%; and finally, the combined VO_2_max and fat mass percent model explained 83.9% of the total variance (all models *P* < 0.05). The equation for the last model is:

**TABLE 5:** Model summary resulting from stepwise multiple regression analysis.

**Independent variable**	**Coefficient**	**St. Error**	***P***	**VIF**	***R*^2^**
*Constant*	3.603.981				0.57
VO_2_max	−26.815	0.7721	0.007	1	
*Constant*	*5.815.224*				*0.6*
vVO_2_max	−348.060	94.362	0.005	1	
*Constant*	407.796				0.595
Fat mass %	141.415	38.818	0.0054	1	
*Constant*	2.039.956				0.839
VO_2_max	−19.001	0.5465	0.0084	1.183	
Fat mass %	102.816	28.255	0.0066	1.183	

*v, velocity; VO_2_, oxygen consumption; VIF, variance inflation factor.*

Race⁢time⁢(min)=203.9956-1.9001×VO⁢max2+10.2816×Fat⁢mass⁢percent (R=20.839,SEE=11.1min,andP=0.0007).

**FIGURE 4:**
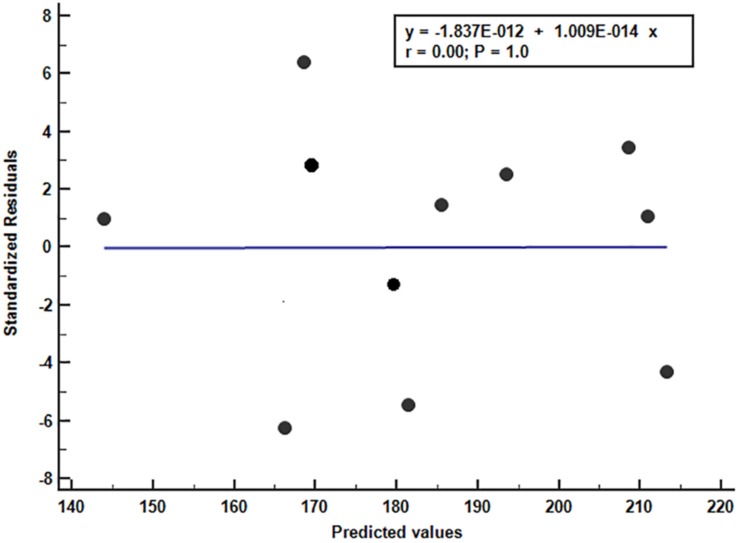
Scatter diagram between residual vs. predicted values with regression line.

The scatter plot of the predicted and residual values of the equation derived from the VO_2_max and fat mass values shows the goodness of fit of this model (Figure 4).

## Discussion

The main objective of this study was to identify the determining physiological variables of short trail running based on the classical model and to improve prediction and if the prediction was improved by adding anthropometric data. The analysis of the results shows an important improvement in the capacity of predicting short trail running performance, with the innovative feature of this study being the inclusion of the variable percentage fat mass.

Several studies in the literature analyze the classical model but explain only 48% of the variance in performance. This prediction is improved to 73.2% when maximum oxygen consumption, evaluation of leg extensor muscle fatigue, and running economy with a 10% slope are included in the multiple regression (Ehrström et al., 2018). In that study, the association between vVO_2_max and race time are identical to that of our study (*r* = −0.75), even with different stress test modalities between studies to reach VO_2_max and therefore also the associated velocity (Morgan et al., 1989; Ehrström et al., 2018) considering that generally the correlations between performance and vVO_2_max have always been well determined in disciplines from 10 to 90 km (Noakes et al., 1990; Billat et al., 2002; Millet et al., 2011). Trail running usually involves challenging and physically demanding uphill running, where runners need to overcome gravity to elevate their body mass as quickly and efficiently as possible and relative VO_2_max is shown to be an important factor for uphill and outdoor running as it expresses the upper limit for aerobic power in relation to body mass (fat mass percent). Both the VO_2_max and fat mass percent are easily obtained in physiological assessments for trail runners.

An important issue are the differences found in the studies concerning exercise protocols applied with different speed and/or slope increments (Vernillo et al., 2017a; Scheer et al., 2018b) and in which, despite these differences, all reach similar levels of VO_2_max (Balducci et al., 2016; Ehrström et al., 2018; Fornasiero et al., 2018) and their associated velocities (vVO_2_max) (Balducci et al., 2016, 2017; Ehrström et al., 2018; Fornasiero et al., 2018; Scheer et al., 2018a). Nonetheless, other studies do find differences in VO_2_max (±2 mL) after the application of different exercise protocols. We believe that all these differences are due to the different levels and aptitudes of the runner, from untrained to trained, elite or highly trained and independent of physiological and biomechanical abilities and neuromuscular adaptation to races with a positive and/or negative slope (Scheer et al., 2018b). Other models that include velocity at VO_2_max explain 47% of the variance in performance of the predictive model (Scheer et al., 2018a). These same authors present a model that includes velocity at the individual anaerobic threshold, as well as the percentage of VO_2_max at the speed of 12 km/h, with a ramp exercise test protocol. For the trail and step models, with several independent variables, they obtain moderate coefficients of determination of 0.68 and 0.65, respectively (Scheer et al., 2018a). Our model, based on VO_2_max alone, contributes 57% of the variance in the prediction of performance and vVO_2_max contributes 60%, although the study samples are very similar in age, BMI, fat mass and certain differences in VO_2_max, which is somewhat higher. The importance of a high VO_2_max value has been associated with a favorable metabolic condition that allows a more efficient use of energy substrates during low-medium intensity exercises in long-distance competitions, in addition to the fact that high VO_2_max values are a beneficial aspect in relation to submaximal intensity and long duration exercise (Millet et al., 2011).

In our case, the associations between performance in mountain races and the variables derived from anthropometry obtained correlation coefficients of *r* = 0.60 and *r* = 0.77 for BMI and fat mass percentage, respectively. However, no associations were found with skeletal muscle mass (*P* = 0.30), with these data being in complete agreement with those of Björklund, regarding the associations with fat mass and fat-free mass measured by dual energy X-ray absorptiometry (DXA) (Björklund et al., 2019). In addition, anthropometric characteristics such as a low fat mass percentage, irrespective of lean mass, are very important for running on hilly terrains with steep slopes.

A study of ultramarathon runners with age and body composition characteristics very similar to our study found no correlations with BMI or fat-free mass, but did find a significant coefficient *r* = 0.55 with fat mass percentage. A combined model derived from anthropometry and graded exercise test of workload of 0.5 W/kg with increments of 0.5 W/kg every 3 min, in which only age and the maximal power in the exercise test are presented as significant model variables, explained only 59% of athletic performance variance (Fornasiero et al., 2018).

Another study associates anthropometric characteristics with performance, but this is a basic study based on weight, height, and BMI all in a large group of athletes of different ages, in which the age subgroup (30–39 years) has higher values for weight, height, and BMI. In addition, this study finds inverse correlations between BMI and average running speed (Hoffman, 2008).

In relation to the variables obtained with the incremental exercise test, significant correlation coefficients for VO_2_max and VO_2_ are found at the aerobic and anaerobic thresholds in an ultramarathon test (*r* = 0.56–0.66), although these correlation coefficients improve (between −0.7 and −0.73) when relating the maximal power and the same powers in VT1 and VT2, all this in watts/kg, signifying the relation to body weight and composition (Fornasiero et al., 2018). In our study, we only found very similar and significant correlation coefficients, higher with VO_2_max and vVO_2_max (both *r* = 0.77), as an expression of the upper limit of aerobic power in relation to body mass and also VO_2_max is presented in two of our models as a significant independent variable. Finally highlight that model derived from VO_2_max and fat mass is not influenced by other variables related to body composition.

### Limitations

The main limitation of this study is that the association between physiological and body composition variables and performance was established in a single competition and, therefore, it is difficult to make comparisons in addition to the small number of participants. It is considered of interest to increase the sample number and to study these relationships in other trail races.

Another area not evaluated in this paper was running economy as one of the factors of the classical model of performance evaluation in endurance runners, although not all authors demonstrate its association with athletic performance (Balducci et al., 2016; Björklund et al., 2019), or other factors such as thigh extensor muscle strength and fatigue indices (Ehrström et al., 2018).

## Conclusion

The variables VO_2_max, vVO_2_max, and fat mass show the highest associations with finishing times. The multiple regression model including VO_2_max and percentage fat mass improves by explained better variance in finishing times.

In agreement with the literature, the classical model is not sufficient to explain performance in short duration trail tests. Additional study factors should be included in the specific physiological assessments. Athletes and coaches may take these results into account to improve performance in these events and to control athletic training.

## Data Availability Statement

The datasets generated for this study are available on request to the corresponding author.

## Ethics Statement

The studies involving human participants were reviewed and approved by the Ethical approval. All the participants were informed of the procedures and signed an informed consent prior to the start of the experimental protocol. The protocol used in this study was approved by the Ethics Committee of the University of Málaga (2013-EMEFYDE-006) and was in accordance with the Declaration of Helsinki (JAMA, 2013). The patients/participants provided their written informed consent to participate in this study.

## Author Contributions

JA-C and VP conceived and designed the study. JA-C, VP, JG, and MC collected the data. JA-C, JG, MC, JB-P, and FO analyzed and interpreted the data, and drafted the manuscript. JA-C, PN, TR, and BK revised the manuscript and approved the final version.

## Conflict of Interest

BK was employed by company Medbase St. Gallen Am Vadianplatz. The remaining authors declare that the research was conducted in the absence of any commercial or financial relationships that could be construed as a potential conflict of interest.

## Footnotes

1 https://www.medcalc.org; 2019
